# Clinical–Functional Evaluation and Test–Retest Reliability of the G-WALK Sensor in Subjects with Bimalleolar Ankle Fractures 6 Months after Surgery

**DOI:** 10.3390/s22083050

**Published:** 2022-04-15

**Authors:** Mario Fernández-Gorgojo, Diana Salas-Gómez, Pascual Sánchez-Juan, David Barbado, Esther Laguna-Bercero, María Isabel Pérez-Núñez

**Affiliations:** 1 Escuelas Universitarias Gimbernat (EUG), Physiotherapy School Cantabria, Movement Analysis Laboratory, University of Cantabria, 39005 Torrelavega, Spain; mario.fernandez@eug.es (M.F.-G.); psanchezjuan@fundacioncien.es (P.S.-J.); mesther.laguna@scsalud.es (E.L.-B.); isabel.perez@unican.es (M.I.P.-N.); 2 International Doctoral School, Rey Juan Carlos University (URJC), 28032 Madrid, Spain; 3 Alzheimer’s Centre Reina Sofia-CIEN Foundation, 28031 Madrid, Spain; 4 Sports Research Centre, Department of Sport Science, Miguel Hernández University of Elche, 03202 Elche, Spain; dbarbado@umh.es; 5 Alicante Institute for Health and Biomedical Research (ISABIAL), 03550 Alicante, Spain; 6 Traumatology Service and Orthopedic Surgery, University Hospital “Marqués de Valdecilla” (UHMV), 39008 Santander, Spain

**Keywords:** malleolar fractures, inertial sensor unit, wearable sensor, walking, spatiotemporal parameters, gait analysis, functional scales, clinical measurement, agreement of measurements

## Abstract

Ankle fractures can cause significant functional impairment in the short and long term. In recent years, gait analysis using inertial sensors has gained special relevance as a reliable measurement system. This study aimed to evaluate the differences in spatiotemporal gait parameters and clinical–functional measurements in patients with bimalleolar ankle fracture and healthy subjects, to study the correlation between the different variables, and to analyze the test–retest reliability of a single inertial sensor in our study population. Twenty-two subjects with bimalleolar ankle fracture six months after surgery and eleven healthy subjects were included in the study. Spatiotemporal parameters were analyzed with the G-WALK sensor. Functional scales and clinical measures were collected beforehand. In the ankle fracture group, the main differences were obtained in bilateral parameters (effect size: 0.61 ≤ *d* ≤ 0.80). Between-group differences were found in cadence, speed, stride length, and stride time (effect size: 1.61 ≤ *d* ≤ 1.82). Correlation was moderate (0.436 < r < 0.554) between spatiotemporal parameters and clinical–functional measures, explaining up to 46% of gait performance. Test–retest reliability scores were high to excellent (0.84 ≤ ICC ≤ 0.98), with the worst results in the gait phases. Our study population presents evident clinical–functional impairments 6 months after surgery. The G-WALK can be considered a reliable tool for clinical use in this population.

## 1. Introduction

Ankle fractures represent 10% of all bone fractures, with bimalleolar or lateral malleolus fractures being the most common according to the selection criteria used in studies [1,2]. The incidence has been increasing over the last two decades to between 71 and 187 fractures per 100,000 people depending on age, sex, and geographic region [3]. Surgical treatment of these fractures is necessary when joint congruence cannot be restored by conservative treatment, as instability, misalignment, and residual displacements will lead to short- and long-term functional impairment [4,5,6,7,8,9].

The importance of the severity of the injury, the surgical intervention, and the immobilization time ranging from 6 to 9 weeks implies significant biomechanical alterations. These consequences are reflected by decreased range of motion of the ankle joint, soft tissue impairments, proprioception, and loss of muscle strength, which indirectly affects functional activities such as walking, balance, jumping, and running [10,11,12,13].

Several studies have reported short- and long-term outcomes after surgery [14,15,16]. A meta-analysis researching the time course of physical recovery after ankle fracture with data from 23 studies concluded that adults, on average, recovered rapidly from activity limitation in the first 3 months after fracture, improved little between 3 and 6 months, and stabilized, without reaching full recovery, at 24 months [5].

Usually, different scores such as the American Orthopedic Foot and Ankle Society Ankle Hindfoot Score (AOFAS) [17] and the Olerud–Molander Ankle Score (OMAS) [18] can be used for the assessment of outcomes after surgery in terms of function and pain. Although these scores can provide a good assessment of function and patient-reported outcome measures (PROMs), they remain quite subjective [19,20].

After an ankle fracture, in addition to assessing functional capacity, it is important to identify clinical parameters that may be conditioning the recovery of these patients. Parameters such as lower extremity strength and range of motion have been studied as good predictors of functional capacity in the short term [10,13]. However, most studies focus on the assessment of ankle strength and do not evaluate other muscle groups of the lower extremity that may be affected after ankle surgery [10,21].

The analysis of spatiotemporal parameters of gait has been widely used to characterize functional performance in different populations [22,23].

This analysis is of particular importance in clinical practice, either to evaluate a rehabilitation process or after surgery [24]. It quantitatively describes the main gait events and thus reflects the patient’s ability to meet the general gait requirements [25]. The most advanced technologies used for gait analysis make use of plantar pressures or 3D motion capture systems to detect changes in gait characteristics; these systems have been validated and are highly reliable for clinical use [26,27,28]. However, despite their advantages, they are expensive and must be operated by specialized personnel. With the advent of inertial measurement systems (IMUs) for spatiotemporal and kinematic assessments came a technological breakthrough in the field of biomechanics, as they are relatively inexpensive and allow the assessment of a virtually unlimited number of steps. In addition, they offer the possibility of assessing gait and movement disorders outside the restricted environments of the clinic and research laboratory [29].

A recent systematic review and meta-analysis provides encouraging results regarding the concurrent validity and reliability of IMUs for measuring step and stride length/time, with small differences depending on their placement on the body. However, measures of spatiotemporal asymmetry present inconsistent results that could be biased by the difference in protocols used for gait analysis or algorithms used for event detection [30]. Based on these results, individual reliability studies of these devices in different populations are needed before recommendations for their clinical use can be made.

Finally, some studies in healthy subjects [31,32] and with lower limb pathology [33] conclude that the individual use of a single IMU placed in the lumbar-sacral spine allows us to obtain reliable information based on trunk acceleration and angular velocity algorithms to estimate the spatiotemporal gait parameters [32,34,35]. Only a small number of studies have focused on gait analysis in patients with ankle malleolar fractures [6,7,8,9,10,19,36,37,38], but to date, none use a single IMU to record these spatiotemporal parameters.

The aims of this study were (1) to evaluate differences in spatiotemporal gait parameters and clinical measures in patients with ankle fracture 6 months after surgery (operated and non-operated ankle) and a control group of healthy subjects, (2) to study the association of gait parameters with clinical measures and functional scales in the ankle fracture group, and (3) to analyze the intra-session test–retest reliability and agreement of measurements from a single inertial sensor, placed on the lumbar-sacral spine, for the spatiotemporal parameters of gait in this population.

## 2. Materials and Methods

### 2.1. Type of Study

This cross-sectional study was carried out in the movement analysis laboratory of the University Schools of Physiotherapy and Speech Therapy Gimbernat-Cantabria attached to the University of Cantabria.

### 2.2. Participants

The population was composed of twenty-two participants (ten women/twelve men) who underwent surgery after a bimalleolar ankle fracture at the Trauma Unit of the University Hospital “Marqués de Valdecilla” (UHMV) in Santander. The surgical technique used was open reduction and internal fixation (ORIF), and the time elapsed from injury to surgery was 4.8 ± 7.6 days. After the immobilization period (3.4 ± 1.2 weeks), progressive and variable rehabilitation was carried out depending on the individual improvement of each case (13 ± 2.4 weeks) 5 days a week by the physiotherapy service of the UHMV. Inclusion criteria were established as 6 months after surgery and age between 18 and 55 years. Patients with previous surgery on the lower limb, bilateral ankle involvement, neurological, and rheumatic pathology were excluded.

Subjects were selected through medical records registered at the UHMV and with the collaboration of the Trauma Unit. After the Informed Consent was approved in writing by the Cantabrian Research Ethics Committee (CEIC) (Reference: 2017.072), they were invited to participate by telephone or email, where they were informed of the objective of the study and the procedure to be followed for its realization.

In this study, we also had a control group (CG) of eleven healthy subjects (six women/five men), consisting of university faculty and staff who agreed to participate on a voluntary basis. These participants were chosen on the basis of characteristics similar to the ankle fracture group in age and sex. All of them were currently free of musculoskeletal pathology of the lower extremity, neurological or rheumatological problems, and with no history of such pathologies.

### 2.3. Procedure

Data collection was carried out in a single individual visit 6 months after surgery, and, after a brief explanation of the procedure to be followed, the Informed Consent was signed. The control group was assessed during the same period as the data collection. Sociodemographic and clinical information regarding the surgery and the rehabilitation process was extracted from the medical records. The clinical data collected were firstly the American Orthopaedic Foot and Ankle Society (AOFAS) Ankle Hindfoot score [39] and the Olerud Molander Ankle Score (OMAS) [18] questionnaires, which assess the functional status of the patients. Subsequently, physical examination of both legs was performed by anthropometric measurement, bimalleolar/calf perimeters, ankle dorsiflexion range of motion (ADF ROM), and hip abductor (ABD)/adductor (ADD) muscle strength. The protocol performed for the clinical measurements was described in detail in our previous study [13].

The gait cycle (GC) analysis was performed with the subject barefoot on a walkway 8 m long and 2.5 m wide where they had to perform 4 laps (32 m) at their normal walking speed. We considered normal speed to be the speed previously preferred by each subject after a brief trial at different speeds following the recommendations of some authors for gait analysis on level ground [40]. Two valid trials were collected for each subject, discarding in the processing the first and last step of each lap. For gait analysis, a wireless inertial sensor system BTS G-WALK (BTS Bioengineering S.p.A., Milan, Italy) weighing 37 g and measuring 70 × 40 × 18 mm was used, placed by means of a semi-elastic belt at the level of the fifth lumbar vertebra (L5) and the first two sacral vertebrae (S1–S2). This inertial system is equipped with 4-Sensor Fusion technology that integrates a triaxial accelerometer (16 bits/axis, ±8 g), a triaxial magnetometer (13 bits, ±1200 uT), a triaxial gyroscope (16 bits/axis, ±250 °/s), and a GPS receiver. All data were collected at a frequency of 100 Hz and transmitted through a Bluetooth 3.0 connection to the computer. A specific software (BTS G-Studio) allows processing the information and calculating the spatiotemporal gait parameters and the percentage of symmetry for these parameters between both legs. The exact algorithms of the G-WALK are unknown and are part of the internal organization of the BTS company. However, some studies validate its use in different populations [34,35,41], although it has not been validated in subjects after ankle fracture.

The general spatiotemporal parameters collected were cadence (strides/min), speed (m/s), stride length (m) (this length was normalized by the length of the legs, trochanter-floor distance), and stride time (s). Bilateral spatiotemporal parameters (leg differences expressed as a percentage of the gait cycle) were step length (% stride length), stance phase (%GC), swing phase (%GC), double support (%GC), single support (%GC), and propulsion index (m/s^2^) (the difference in anterior/posterior acceleration of the body barycenter during the single support phase of the right and left side’s gait cycle) [30].

### 2.4. Statistical Analysis

First, participants in the ankle fracture group (AFG) were classified according to their operated and non-operated ankle. For the CG, the dominant leg was taken as the reference. Sociodemographic and clinical variables were described. For categorical variables, percentages with their corresponding 95% confidence intervals (95%CI) were estimated, and for continuous variables, means were estimated with their standard deviation or, if they did not follow a normal distribution, their median and range. The Shapiro–Wilk test was performed to analyze the normality of the variables.

In the AFG, the results of the different variables were obtained for both ankles (operated/non-operated). The difference between them was analyzed using Student’s *t*-test for paired samples (expressed as mean difference) or its non-parametric equivalent Wilcoxon matched-pairs signed-ranks test (expressed with the Z-value typed for comparison with that of a standardized normal distribution). Differences between groups (AFG/CG) were performed using the Student’s *t*-test for independent samples or its equivalent non-parametric Mann–Whitney U test. Likewise, the effect size was calculated using Cohen’s *d* or Hodges’ *g*, whose values are quantified as follows: 0.2 small, 0.5 medium, and 0.8 large [42].

The relationship between clinical measurements and functional scales with spatiotemporal gait parameters was analyzed using Pearson’s correlation coefficient (r) or Spearman’s rank correlation (Rho) (non-parametric). A regression model (simple and multiple linear regression, r^2^), expressed together with the value of the F-statistic, was then applied to the variables that showed a significant correlation to determine the extent to which clinical measurements or functional scale scores could predict the results of the gait analysis. Intra-session test–retest reliability of spatiotemporal gait parameters measured with the G-WALK in the AFG was calculated using two valid trials. For relative reliability, an ICC_2,1_ model with a 95% CI was used following the recommendations described in the literature [43]. The ICC values were classified as follows: excellent (0.90 to 1.00), high (0.70 to 0.89), moderate (0.50 to 0.69) and low (<0.50) [44]. Absolute reliability was obtained with the standard error of measurement (SEM) calculated as SEM = SD × √(1 − ICC) [45]. The SEM values were expressed in the same units as the mean value and in a percentage (SEM%) to facilitate interpretation and extrapolation of the results to other individuals.

Finally, Bland–Altman plots analysis with 95% limits of agreement (LoA; mean differences: ±1.96 SD) were generated to visualize the degree of agreement between the measurements reported. Systematic error (bias) was obtained using the mean of the differences.

Statistical analysis of the data was performed using SPSS 20.0 software (Statistical Product and Service Solutions IBM SPSS Statistics 19.0 2010).

## 3. Results

A total of twenty-two patients with bimalleolar ankle fractures and 6 months after surgery participated in the present study. The mean age was 43.5 ± 10.2 years, with ages ranging from 21 to 55 years. Eleven healthy subjects with a mean age of 39.9 ± 8.6 were in the control group (CG). Table 1 describes the demographic and anthropometric characteristics of both groups, as well as the functional status of the AFG.

The difference between the operated and non-operated ankle in the spatiotemporal gait parameters showed a significant difference in step length (−3.8%; *p* = 0.009; *d* = 0.61), stance phase (Z = −2.9; *p* = 0.004; *g* = 0.76), swing phase (Z = −2.9; *p* = 0.004; *g* = 0.76), single support (Z = −3.0; *p* = 0.002; *g* = 0.80), and propulsion index (−0.8 m/s^2^; *p* = 0.010; *d* = 0.62). We also found differences in clinical measurements except for ADD strength with an effect size between 0.15 ≤ *d* ≤ 2.30 (Table 2).

In the comparative analysis between AFG and CG of spatiotemporal gait parameters (Table 3), we found a significant difference and a high effect size in cadence (−13.8 p/m; *p* < 0.001; *d* = 1.61); speed (−0.24 m/s; *p* < 0.001; *d* = 1.71), stride length (−0.18 m; *p* = 0.003; *d* = 1.82); stride time (0.16 s; *p* < 0.001; *d* = 1.65); single support (−3.0%; *p* = 0.045; *d* = 0.71), and propulsion index (−1.7 m/s^2^; *p* = 0.013; *d* = 0.98). The differences found in clinical measurements were significant for bimalleolar perimeter (3.2 cm; *p* < 0.001; *d* = 1.64), ADF ROM (−19.1°; *p* < 0.001; *d* = 2.71), and ABD strength (−8.6%; *p* = 0.005; *d* = 1.12).

Correlation analysis between clinical measurements and spatiotemporal gait parameters in the operated ankle showed statistically significant results and a moderate to large effect size (Table 4). Regression model analysis showed that both ADF ROM, ABD strength, and calf perimeter scores can explain the variability of gait analysis results between 20% and 46%. Specifically, cadence increased with increasing ADF ROM r = 0.552 (F (1, 21) = 8.7, r^2^ = 0.30, *p* = 0.009); speed increased with increasing ADF ROM r = 0.533 and increasing ABD strength r = 0.436 (F (1, 21) = 6.6, r^2^ = 0.25, *p* = 0.018); stride length increased with increasing ABD strength r = 0.444 (F (1, 21) = 4.9; r^2^ = 0.20); stride time decreased with increasing ADF ROM r = −0.554 (F (1, 21) = 8.8; r^2^ = 0.26); propulsion index was greater the higher the ADF ROM r = 0.523 and calf perimeter r = 0.447 (F (1, 21) = 10, r^2^ = 0.46, *p* = 0.001). Finally, with respect to the AOFAS scores, the correlation was positive with cadence (r = 0.540), speed (r = 0.428) and stride time (r = 0.547). Simple linear regression analysis showed that the AOFAS score could only explain the variability of cadence (F (1, 21) = 8.2, r^2^ = 0.29, *p* = 0.009) and stride time (F (1, 21) = 8.5, r^2^ = 0.30, *p* = 0.008) by 30%.

The intra-session test–retest reliability analysis, including ICC_2,1_, SEM, and SEM% values, are shown in Table 5. Excellent relative reliability scores (0.95 ≤ ICC ≤ 0.98) were found for the general parameters of gait analysis, as well as low absolute reliability values between 1.56% ≤ SEM% ≤ 2.47%. For the bilateral parameters, a good to excellent ICC score was found with values between 0.84 and 0.95. The worst SEM% values were for double support (11.20%) in the operated ankle and propulsion index (7.88%) in the non-operated ankle.

**Figure 1:**
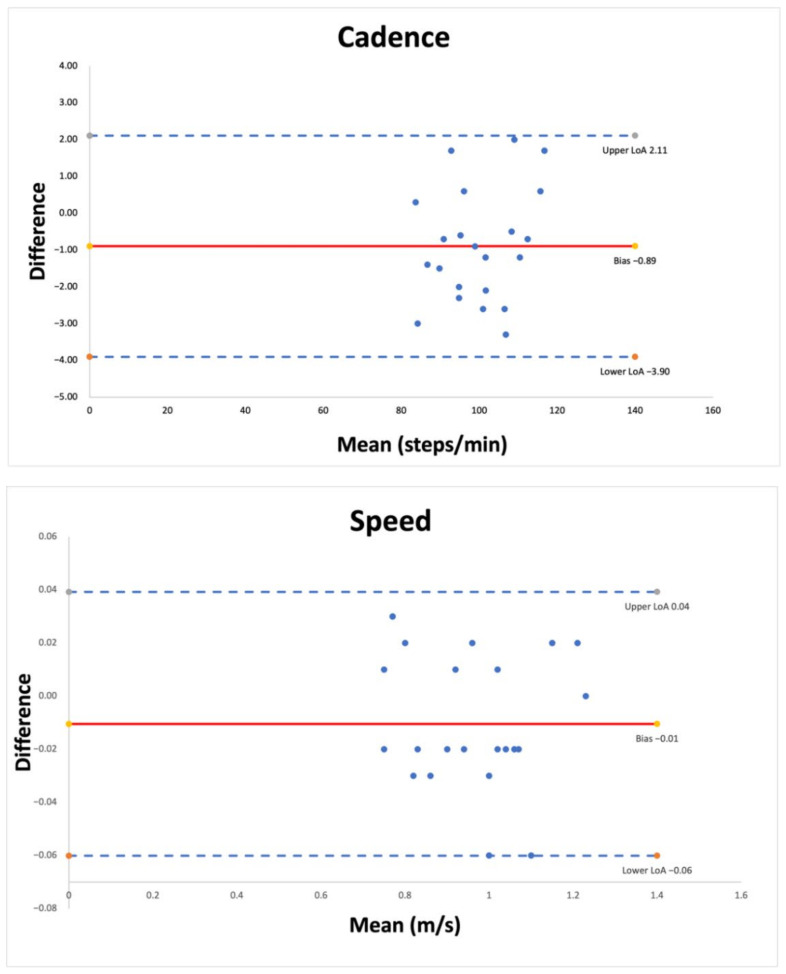
Bland-Altman plots for cadence and speed. Each graph presents the mean difference (solid line) and 1.96-fold standard deviation of difference (dashed line) indicating the limits of agreement between the measurement.

**Figure 2:**
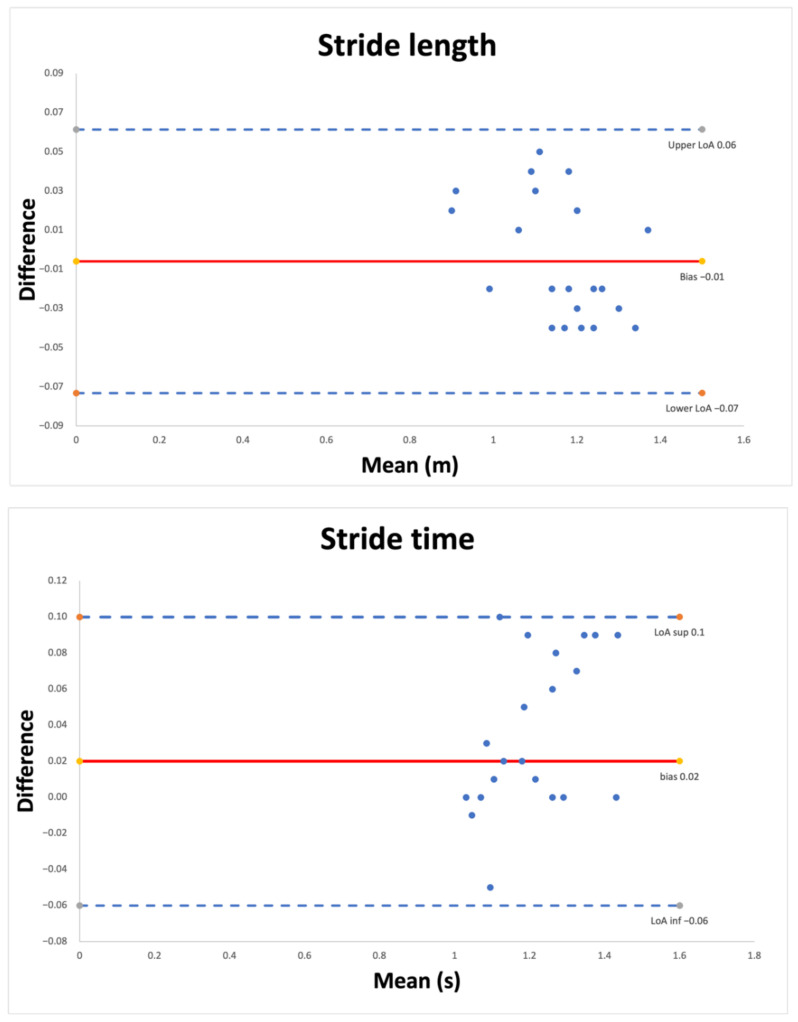
Bland-Altman plots for stride length and stride time. Each graph presents the mean difference (solid line) and 1.96-fold standard deviation of difference (dashed line) indicating the limits of agreement between the measurement.

Figure 1 and Figure 2 show the Bland–Altman plots comparing the results of the spatiotemporal gait parameters. The horizontal line represents the mean of the differences, while the dotted lines represent the confidence interval. The Bland–Altman plot analysis showed an excellent degree of agreement between measurements for speed (bias = −0.01; LoA = −0.06; 0.04) and stride length (bias = 0.01; LoA = −0.07; 0.06). Single support (bias = −1.79; LoA = −9.21; 6.63) in the operated ankle and double support (bias = 1.13; LoA = −5.57; 7.83) in the non-operated ankle showed the lowest degrees of agreement. The mean error and limits of agreement for the remaining variables are reported in Table 5.

## 4. Discussion

One of the aims of our study was to evaluate the spatiotemporal gait parameters in patients with bimalleolar ankle fractures 6 months after surgery and compare them with healthy subjects.

In the AFG, we found a clear difference between both legs in the gait phases. In particular, the double support was the only parameter where no differences were obtained. Regarding the comparative analysis with the CG, the main differences were obtained in cadence, speed, stride length, stride time, and single support in the operated ankle. Our results are in agreement with a study by Suciu et al. [37] in thirty patients with bimalleolar ankle fractures and twenty-one healthy subjects, in which they found differences between both ankles in step time, step length, swing phase, stance phase, and single support after 12 weeks of specific rehabilitation. Compared to the control group they found differences in stride length and speed. However, in contrast to our findings, they found no differences in cadence or stride time. Aspects that may determine the differences between the two studies include the measurement system used and the type of rehabilitation performed. On the other hand, half of the participants in their study were over 50 years of age, and the results were considerably different from younger adults whose recovery process was very rapid. Another study conducted on patients with trimalleolar ankle fractures 6 months after surgery found similar results to ours when compared to healthy subjects in speed, cadence, stride length, and stride time [46]. In contrast, our patients with bimalleolar fractures had a stride length 18 cm longer than that obtained in their study, which we believe to be a clinically important difference. However, other studies conclude that there are no short-term differences in gait characteristics between bimalleolar and trimalleolar ankle fractures. [6,7]. Segal et al. [7], in their study of forty-one subjects with ankle fracture and seventy-two healthy subjects, found in the bimalleolar fracture group (*n* = 15) differences between the two ankles in step length (−29.2% SL) and single support (−15.9% GC). In our patients, we also found this asymmetry in step length (−3.8% SL) and single support (−4.1% GC), although the difference was not as large. In addition, the speed was only 0.48 m/s, very different from what we found (0.94 m/s). The differences in the results seem to be justified by the period of the measurements, as the Segal et al. study was performed from the 12th week after surgery, just when weight-bearing on the operated ankle was allowed.

As we have just seen, the decrease in speed and stride length is very much conditioned by the time of recovery in which the patients find themselves. In this sense, another study carried out at the beginning of the rehabilitation process on twenty-four patients with ankle fractures and twenty-four healthy controls found a difference between groups of −40 cm in stride length, greater than the difference found in our study [6]. In contrast, if we look at what happens in the long term, some authors find that even one year after surgery, the spatiotemporal gait parameters are not yet normalized. In particular, gait speed is significantly lower in patients with malleolar fractures compared to healthy subjects [8,38]. Other authors, however, only found a reduced gait speed but did not consider this to be clinically relevant [9].

Specific and individualized rehabilitation is fundamental in ankle recovery, and no less important is to keep a functional and clinical record throughout the recovery process [47]. A good way to estimate the patient’s functional capacity is by assessing ankle mobility or lower extremity strength. These clinical parameters allow prediction of performance in functional tasks such as gait [10].

In this regard, in our work, we evaluated ADF ROM, ABD/ADD hip muscle strength, and bimalleolar/calf perimeter in both study groups and additionally studied the degree of correlation, including regression models, between-gait parameters, and clinical measurements in the AFG.

ADF ROM is one of the most studied variables after ankle injury [48]. Despite rehabilitation efforts to improve ankle motion, short- and medium-term studies conclude that the gain is only 6–12% [48]. Such a low gain in range of motion is a major barrier to acquiring pre-injury status. Some authors put a cut-off point of 30° of weight-bearing dorsiflexion as the minimum to be able to perform tasks such as descending stairs or squatting without problems [15]. In our study, we found in the AFG a difference between the operated and non-operated ankle in the ADF ROM of −12.7° and −19.1° concerning the CG. Using a measurement methodology similar to ours, Nilsson et al. 2009 [36], in a sample of 105 patients with ankle fractures 6 months after surgery, obtained similar results to ours. In our work, the measurement was performed before the gait analysis to correlate it with the different spatiotemporal parameters. This ankle motion quantification system obtained excellent intra- and inter-test reliability [49]. Studies analyzing the kinematics of gait show the restrictions of the ADF ROM during the different phases of gait [9,38,50]; however, they do not relate this decrease in the movement to the spatiotemporal parameters. In our study, we were able to observe how ankle ADF ROM and ABD strength had a moderate association with cadence, speed, stride length, and stride time; moreover, they predicted up to 30% of the variability of their values.

The muscle atrophy observed in the calf perimeter could influence the gait pattern of patients with ankle fractures. In our study, only 24 weeks after surgery, the calf musculature of the operated ankle had a smaller calf perimeter (−1.3 cm; *p* = 0.002) compared to the non-operated ankle. Human studies quantifying the effect of disuse on muscle morphology show that in only 8 weeks of immobilization, the cross-sectional area measured with MRI shows decreases of 19% and 24% in the anterior and posterior calf muscle compartments [51]. The strength and activation of the plantar flexor muscles also suffer a significant loss [52], and their improvement after a period of rehabilitation has already been studied [10,36]. In our research, we did not directly assess the strength of the plantar flexors; however, we analyzed the propulsion index that could be associated with the strength of this muscle group during single support [53]. In reference to this parameter, we found that the propulsion index was significantly lower (−0.8 m/s^2^) when comparing the operated and non-operated ankle of the AFG, and even lower (−1.7 m/s^2^) when compared to the CG. Furthermore, we found a positive and significant correlation between calf perimeter and propulsion index (r = 0.447), and together with ADF ROM (r = 0.523), it could predict 46% of the propulsion index score. These results reflect the importance of having a good range of motion and calf muscle volume to be able to propel yourself adequately during gait.

Among the questions raised before conducting this study were the consequences that non-weight-bearing immobilization after surgery might have on the hip musculature. In this regard, there is a lack of studies identifying this impact on gait, although it has been studied in dynamic balance [13]. In the present study, we found a significant difference in ABD strength of both legs within and between groups. Furthermore, the correlation was positive and moderate with speed (r = 0.436), stride length (r = 0.444) and individual support (r = 0.491). Despite these results, ABD strength alone was not a significant predictor of any of the gait parameters. Based on these results, we could think that, despite the ABD strength deficit, gait is not a task that requires the recruitment of this musculature as balance or running could be.

Quantitative information on the evolution of recovery of physical function after an ankle fracture is essential for adequate patient care. Professionals can make use of prognostic data to understand the course of recovery and make the right decisions throughout the rehabilitation process. In our study where we assessed functional condition using the OMAS and AOFAS scales, we found that 6 months after surgery, patients still had pain and impaired function. A meta-analysis studying the short-, medium-, and long-term prognosis of function improvement in patients operated on after an ankle fracture tells us that improvement in the first 6 months is rapid but incomplete, with only 78% of function recovered [5]. In our research, we even found worse results on the OMAS subjective functional scale (57.3 ± 22.0), which was used in most of the studies included in the meta-analysis. The reason for this low score could lie in the subjectivity of the scale due to its characteristics or the state of health of the patients at the time of surgery [54]. We obtained better results on the AOFAS scale (73.6 ± 11.4); in addition, we found that a higher cadence (r = 0.540) and speed (r = 0.428), as well as a shorter stride time (r = −0.547), were moderately correlated with better scores. However, this correlation was not found with the OMAS scale. This is in line with the results of other studies in which this correlation also did not exist [37] or was weak [8,9].

The final aim of this study was to assess the test–retest reliability and agreement of measurements from a single inertial sensor, placed on the lumbar-sacral spine, in this population.

In our results, we found high to excellent intra-session test–retest reliability, with ICC scores between 0.84 and 0.98; the worst ICC values were obtained for the variables single support and double support at both ankles. Our results are consistent with those found by De Ridder et al. [35] in a group of thirty healthy subjects, in which they obtained high to excellent reliability values (0.84 ≤ ICC ≤ 0.99) for the spatiotemporal gait parameters after five valid trials. In line with our findings and those of Rider et al., another study conducted on a large healthy population and with different neurological pathologies, finds ICC values between 0.82 and 0.97, with the worst result for the stance and swing phase [55]. Despite the similarities in our results, the two previous studies only assess relative reliability but do not provide data on absolute reliability that would allow us to see the degree of variation of repeated measurements in individuals. In our investigation, the lowest SEM was obtained for stride length (0.02 m), which represents only 1.56% of the SEM%. Furthermore, the degree of agreement between the two trials was excellent for speed, stride length, and stride time, with bias values close to 0 and a small range in LoA. Bravi et al. [33], on a sample of twenty subjects with lower limb pathology, found moderate to excellent inter-rater reliability (0.59 ≤ ICC ≤ 0.95), with the lowest values corresponding to the phases of the gait cycle. In our work, we did not find such low ICC values in the gait phases; however, we found a higher SEM% in double support and propulsion index of both ankles, although they represent less than 11% of the SEM%. Concerning the limits of agreement and estimated bias, the single support of the operated ankle obtained the worst accuracy (bias = −1.79; LoA = −9.21; 5.63), probably due to the presence of three outliers. Bravi et al. justify the low reliability of gait phase recognition to the reduced pelvic motion in their study population. This is in agreement with that described by Zijlstra and Hof [31], who report the influence of the pelvis in differentiating normal and pathological gait patterns. In relation to this, the differences between our study population and the characteristics of the Bravi et al. sample, with subjects who have undergone hip or knee replacement with subjects having undergone hip or knee repositioning and possibly presenting greater functional limitations, may justify the differences obtained.

Based on our results and the previously mentioned studies in different populations, it seems to indicate that the estimation of gait phases may be affected by an asymmetric gait cycle. Gait speed is another point to consider, as it has been shown to be a parameter that affects the validity of wearable sensors [56]. However, the ICC and SEM results obtained in our research, as well as the low bias values and limits of agreement, we consider to be within clinically acceptable limits.

Our study has some limitations. Firstly, the characteristics of a cross-sectional study. However, we believe that this type of study, carried out 6 months after surgery, is necessary because of the importance of an objective and global identification of functional problems that can guide a more specific rehabilitation. Secondly, we have a small sample size for the control group. However, the sample size calculation based on the differences in gait speed between groups indicates adequate power to detect a minimal clinically important difference. Finally, in our research, we did not use a gold-standard system for the concurrent validity of the G-WALK. Although it has not been studied in patients with ankle fractures, there are already studies in different populations where moderate to excellent levels of agreement and reliability were obtained, with the lowest values corresponding to the gait phases [33,34,35,41].

## 5. Conclusions

In our sample of patients with bimalleolar ankle fracture, 6 months after surgery, the analysis of the spatiotemporal gait parameters shows a clear asymmetry between both legs in the different gait phases. Furthermore, compared to healthy subjects, there is a decrease in cadence, speed, and stride length, as well as an increase in stride time. The decrease in clinical parameters such as ADF ROM, ABD hip muscle strength, and calf perimeter influence gait performance and may even explain 20–46% of the results in certain gait parameters. The low scores obtained at 6 months on the AOFAS and OMAS scales reveal a slow recovery of function and symptomatology; furthermore, better scores on the AOFAS scale are associated with better cadence and stride time. Finally, test–retest reliability and agreement analysis of the measurements made with the G-WALK sensor shows good to excellent results in our study population. Therefore, it can be considered a reliable gait analysis system, and its use could be justified in the clinical setting, although being cautious with the interpretation of the results in the identification of gait phases.

## Figures and Tables

**Table 1:** Demographic, anthropometric, and functional characteristics of patients with bimalleolar ankle fractures 6 months after surgery and the control group.

Type (*n* = 22)	AFG (*n* = 22) Mean ± SD	95%CI	CG (*n* = 11) Mean ± SD	95%CI
Age (years)	43.5 ± 10.2	39.0; 48.0	39.9 ± 8.6	34.1; 45.7
Sex Women (%); Men (%)	45% (W); 55% (M)		55% (W); 45% (M)	
Height (cm)	169.3 ± 9.5	164.8; 173.7	170.5 ± 7.9	165.2; 175.8
Weight (kg)	77.8 ± 10.6	73.1; 82.5	74.0 ± 9.1	67.9; 80.1
Operated Limb Length	85.6 ± 5.9	82.9; 88.2	86.2 ± 5.5 *	82.6; 89.9 *
Healthy Limb Length (cm)	85.6 ± 5.9	82.9; 88.2		
Days from injury to surgery	4.8 ± 7.6	1.4; 8.1		
Immobilization (weeks)	3.4 ± 1.2	2.8; 3.9		
AOFAS Ankle Hindfoot score	73.6 ± 11.4	71.9; 75.3		
OMAS	57.3 ± 22.0	54.1; 60.6		

AFG: ankle fracture group; CG: control group; SD: standard deviation; CI: confidence interval; AOFAS: American Orthopedic Foot and Ankle Society; OMAS: Olerud Molander Ankle Score; Dominant leg CG * cm.

**Table 2:** Difference between the operated and non-operated ankle in clinical measurements, spatial–temporal gait parameters, and dynamic plantar pressure.

	Type (*n* = 22)	Operated Ankle Mean ± SD/Median (Range)	Non-Operated Ankle Mean ± SD/Median (Range)	Differences between Ankles Mean (95% CI)/Z ^1^	Cohen’s *d*/Hedges’ *g*	*p* Value *
Clinical measurements	Calf perimeter (cm)	34.2 ± 4.0	35.5 ± 4.4	−1.3 (−2.0; −0.5)	0.78	0.001 *
	Bimalleolar perimeter (cm)	25.1 ± 2.1	24.1 ± 2.1	1.0 (0.8; 1.2)	2.30	<0.001 *
	ADF ROM (degrees)	22.8 ± 7.7	35.4 ± 5.3	−12.7 (−15.1; −10.3)	2.23	<0.001 *
	Strength ABD (%)	25.5 ± 7.2	29.3 ± 8.6	−3.8 (−6.4; −1.2)	0.62	0.006 *
	Strength ADD (%)	26.3 ± 9.1	25.8 ± 8.6	0.6 (−1.1; −2.2)	0.15	0.491
Spatiotemporal parameters	Cadence (step/min)	99.9 ± 9.8				
	Speed (m/s)	0.94 ± 0.1				
	Stride length (m)	1.28 ± 0.1				
	Stride time (s)	1.21 ± 0.1				
	Step length % SL	48.1 ± 3.1	51.9 ± 3.1	−3.8 (−6.7; −1.1)	0.61	0.009 *
	Stance % GC ^1^	63.4 (20.3)	67.4 (17.9)	−2.9	0.76	0.004 *
	Swing % GC ^1^	36.6 (20.3)	32.6 (17.9)	2.9	0.76	0.004 *
	Double support % GC	15.0 ± 4.3	16 ± 2.1	−1.0 (−2.8; −0.8)	0.25	0.267
	Single support % GC ^1^	32.6 (17.6)	36.7 (20.6)	−3.0	0.80	0.002 *
	Propulsion index (m/s^2^)	5.2 ± 1.8	6.0 ± 1.4	−0.8 (−0.2; −1.2)	0.62	0.010 *

SD: standard deviation; CI: confidence interval; ADF ROM: ankle dorsiflexion range of movement; ABD: hip abductor muscle (normalized by body mass); ADD: hip adductor muscle (normalized by body mass); ROM: range of movement; GC: gait cycle; SL: stride length; Cohen’s *d*: size effect; Hedges’ *g*: size effect (non-parametric); ^1^ Wilcoxon matched-pairs signed-ranks test (non-parametric; expressed with the typed Z-value); * Significance level *p* < 0.05.

**Table 3:** Difference between bimalleolar ankle fracture patients and the control group in clinical measurements and spatiotemporal gait parameters.

	Type	AFG (*n* = 22) Mean ± SD/Median (Range)	CG (*n* = 11) Mean ± SD/Median (Range)	Differences between Ankles Mean (95% CI)/Z ^1^	Cohen’s *d*/Hedges’ *g*	*p* Value *
Clinical measurements	Calf perimeter (cm)	34.2 ± 4.0	33.7 ± 2.5	0.5 (3.1; −2.3)	−0.14	0.76
	Bimalleolar perimeter (cm)	25.1 ± 2.1	21.9 ± 1.6	3.2 (4.6; 1.7)	−1.64	<0.001 *
	ADF ROM (degrees)	22.8 ± 7.4	41.9 ± 6.1	−19.1 (−13.8; −24.4)	2.71	<0.001 *
	Strength ABD (%)	25.5 ± 7.2	34.2 ± 8.8	−8.6 (−2.7; −14.5)	1.12	0.005 *
	Strength ADD (%)	26.3 ± 9.1	32.7 ± 9.2	−6.4 (0.5; −13.2)	0.72	0.06
Spatiotemporal parameters	Cadence (step/min)	99.9 ± 9.8	113.7 ± 5.2	−13.8 (−8.4; −19.1)	1.61	<0.001 *
	Speed (m/s)	0.94 ± 0.1	1.18 ± 0.2	−0.24 (−0.12; −0.36)	1.71	<0.001 *
	Stride length (m)	1.28 ± 0.1	1.46 ± 0.1	−0.18 (−0.06; −0.27)	1.82	0.003 *
	Stride time (s)	1.21 ± 0.1	1.05 ± 0.1	0.16 (0.23; 0.08)	−1.65	<0.001 *
	Step length % SL	48.1 ± 3.1	49.2 ± 1.2	−1.1 (0.6; −2.8)	0.42	0.196
	Stance % GC ^1^	63.4 (20.3)	63.6 (9.5)	−0.2	0.03	0.834
	Swing % GC ^1^	36.6 (20.3)	36.4 (10.3)	−0.4	−0.02	0.688
	Double support % GC	15.0 ± 4.3	14.3 ± 3.3	0.7 (−2.3; 3.7)	−0.17	0.612
	Single support % GC	32.6 ± 4.5	35.6 ± 3.6	−3.0 (−0.1; −6.2)	0.71	0.045 *
	Propulsion index (m/s^2^)	5.2 ± 1.8	6.9 ± 1.6	−1.7 (−1.1; −2.3)	0.98	0.013 *

AFG: ankle fracture group; CG: control group; SD: standard deviation; CI: confidence interval; ADF ROM: ankle dorsiflexion range of movement; ABD: hip abductor muscle (normalized by body mass); ADD: hip adductor muscle (normalized by body mass); ROM: range of movement; GC: gait cycle; SL: stride length; Cohen´s *d*: size effect; Hedges’ *g*: size effect (non-parametric); ^1^ Wilcoxon matched-pairs signed-ranks test (non-parametric; expressed with the typed Z-value); * Significance level *p* < 0.05.

**Table 4:** Correlation between clinical measurements and functional scales with the spatiotemporal gait parameters in operated ankle.

	Clinical Measurements and Functional Scales					
Spatiotemporal Gait Parameters	ADF ROM	Strength ABD	Bimalleolar Perimeter	Calf Perimeter	AOFAS	OMAS
Cadence (step/min) ^1^	0.552 **	0.405	0.230	0.177	0.540 **	0.415
Speed (m/s) ^1^	0.533 *	0.436 *	0.335	−0.124	0.428 *	0.247
Stride length (m) ^1^	0.413	0.444 *	0.070	−0.289	0.247	0.083
Stride time (s)	−0.554 **	−0.393	−0.263	−0.205	−0.547 **	−0.398
Step length % SL ^1^	−0.001	0.231	0.056	−0.144	0.163	0.205
Stance % GC ^2^	−0.054	−0.178	−0.112	0.144	0.115	0.172
Swing % GC ^2^	0.054	0.178	0.112	−0.144	−0.115	−0.172
Double support % GC ^1^	−0.224	−0.303	−0.060	0.222	−0.069	0.036
Single support % GC ^2^	0.318	0.491 *	−0.001	−0.076	0.402	0.284
Propulsion index (m/s^2^) ^1^	0.516 *	−0.052	0.122	0.449 *	0.407	0.261

^1^ Pearson’s correlations (r); ^2^ Spearman’s rank correlation coefficient (Rho) (non-parametric); * *p* < 0.05; ** *p* < 0.01.

**Table 5:** Intra-session test–retest reliability spatiotemporal gait parameters with the G-WALK sensor. Limits of agreement (Bland–Altman analysis) and mean of the differences (bias) between two trials.

	Spatiotemporal Gait Parameters	ICC (95%CI)	SEM (95% CI)	SEM%	LoA (Lower; Upper)	Bias
	Cadence (step/min)	0.95 (0.89; 0.97)	2.21 (0.79; −3.64)	2.21	−3.91; 2.12	−0.89
	Speed (m/s)	0.97 (0.93; 0.98)	0.02 (0.01; 0.05)	2.12	−0.06; 0.04	−0.01
	Stride length (m)	0.98 (0.97; 0.99)	0.02 (0.01; 0.03)	1.56	−0.07; 0.06	0.01
	Stride time (s)	0.95 (0.70 0.98)	0.03 (0.01; 0.05)	2.47	−0.06; 0.10	0.02
Operated Ankle	Step length % SL	0.90 (0.82; 0.94)	1.01 (0.55; 1.46)	2.09	−2.17; 1.92	−0.12
	Stance phase % GC	0.91 (0.84; 0.94)	1.43 (0.75; 2.12)	2.25	−5.02; 4.51	−0.26
	Swing phase % GC	0.86 (0.75; 0.91)	1.79 (1.10; 2.47)	4.89	−4.51; 5.02	0.26
	Double support % GC	0.85 (0.74; 0.91)	1.68 (1.06; 2.31)	11.20	−6.80; 7.74	0.47
	Single support % GC	0.84 (0.74; 0.91)	1.82 (1.17; 2.48)	5.58	−9.21; 5.63	−1.79
	Propulsion index (m/s^2^)	0.90 (0.83; 0.94)	0.45 (0.24; 0.65)	7.50	−1.79; 1.89	0.05
Non-operated Ankle	Step length % GC	0.90 (0.84; 0.95)	1.01 (0.55; 1.46)	1.94	−1.92; 2.17	0.12
	Stance phase % GC	0.94 (0.89; 0.96)	1.12 (0.46; 1.77)	1.66	−2.96; 4.50	0.77
	Swing phase % GC	0.92 (0.86; 0.95)	1.29 (0.63; 1.95)	3.95	−4.50; 2.96	−0.77
	Double support % GC	0.84 (0.73; 0.90)	1.06 (0.68; 1.44)	6.62	−5.57; 7.83	1.13
	Single support % GC	0.84 (0.73; 0.91)	1.90 (1.22; 2.58)	5.17	−7.59; 8.17	0.29
	Propulsion index (m/s^2^)	0.95 (0.92; 0.97)	0.41 (0.15; 0.68)	7.88	−1.89; 1.20	−0.34
	Propulsion index (m/s^2^)	0.95 (0.92; 0.97)	0.41 (0.15; 0.68)	7.88	−1.89; 1.20	−0.34

CI: confidence interval; ICC: intraclass correlation coefficient; SEM: standard error of the measurement; LoA: limits of agreement; Bias: mean of the differences.

## Data Availability

Not applicable.
